# Efficiency divergence and convergence in China’s hospital sector: empirical insights from Guangdong Province using a multi-method approach

**DOI:** 10.3389/fpubh.2025.1651744

**Published:** 2025-09-11

**Authors:** Jianxin Yu, Yongyi Xu, Jindong Wu, Baoling Wu, Zijuan Zhang, Weizhang Huang, Hanxiang Gong

**Affiliations:** ^1^The First Affiliated Hospital, Guangzhou Medical University, Guangzhou, Guangdong, China; ^2^The Second Affiliated Hospital, Guangzhou Medical University, Guangzhou, Guangdong, China; ^3^Stomatological Hospital of Southern Medical University, Guangzhou, Guangdong, China

**Keywords:** Guangdong Province, healthcare institutions, service efficiency, tertiary public hospitals, performance evaluation

## Abstract

**Background:**

Since the launch of China’s new round of healthcare system reforms in 2009, improving service efficiency has become a critical focus for enhancing the equitable allocation of medical resources and overall healthcare quality. Guangdong Province, as one of China’s most economically dynamic regions, faces significant challenges in addressing disparities in hospital service efficiency and optimizing resource utilization.

**Objective:**

This study aims to comprehensively analyze the differences, dynamic evolution, and influencing factors of service efficiency among healthcare institutions in Guangdong Province. The goal is to provide scientific evidence for narrowing efficiency gaps between hospitals, enhancing overall service quality, and informing policy development.

**Methods:**

A comprehensive evaluation system for hospital service efficiency in Guangdong Province was constructed. Data Envelopment Analysis (DEA) was employed to measure efficiency levels. The Dagum Gini coefficient decomposition method was used to examine the sources of efficiency disparities among different hospital categories. Kernel density estimation was employed to investigate the dynamic distribution of service quality, while the Tobit regression model was used to identify key factors influencing healthcare service efficiency.

**Results:**

The findings indicate that significant differences in service efficiency existed among various categories of hospitals in Guangdong Province from 2018 to 2022. Specialty hospitals demonstrated the highest average overall efficiency, whereas general hospitals recorded the lowest efficiency and growth rates. The performance of healthcare institutions in terms of pure technical efficiency, scale efficiency, and overall efficiency showed fluctuations, but a general trend of recovery was observed. The Dagum Gini coefficient decomposition revealed a relatively high degree of internal efficiency inequality within general hospitals, peaking in 2021, reflecting heterogeneity in scale, management, and resource allocation. The Gini coefficient between specialty and general hospitals was also comparatively high. Kernel density estimation indicated a bimodal distribution in service efficiency, highlighting the heterogeneity of efficiency levels and an increase in hospital categories with lower service quality. The Tobit model analysis confirmed that a lower mortality rate among low-risk cases had a significant positive impact on service efficiency, while average appointment rate, the proportion of health technicians, and the proportion of senior health technicians had significant negative effects.

**Conclusion:**

It is recommended to deepen reforms of internal governance structures and enhance cross-level collaboration within healthcare institutions. Establishing efficiency-oriented mechanisms, optimizing human resource management, strengthening departmental collaboration, and leveraging information technology can effectively improve the service efficiency of healthcare institutions in Guangdong Province.

## 1 Introduction

Since 2009, the Chinese government has initiated a new wave of healthcare system reforms, focusing on equitable medical resource allocation, enhancing service efficiency and quality, and consistently improving the performance evaluation framework for public hospitals (1, 2). The “Healthy China 2030” Planning Outline emphasizes a people-centered approach, proposing to enhance health governance by improving the basic healthcare service system, optimizing resource allocation, advancing the integration of medical care and prevention, and strengthening quality supervision (3). These policies have catalyzed the introduction of innovative measures nationwide, including hierarchical diagnosis and treatment systems, medical alliances, and hospital group formations. These initiatives are intended to alleviate the issues of “difficult and expensive access to healthcare,” and to promote the equalization and universality of basic public healthcare services (4). At the same time, the national performance evaluation for tertiary public hospitals has become a pivotal mechanism in China’s deepening healthcare reform, serving as a core tool for measuring the comprehensive service capacity, operational efficiency, and quality and safety of medical institutions, and facilitating the transformation of hospital governance toward greater efficiency, scientific management, and standardization (5).

Despite these efforts, significant disparities in service efficiency persist among different regions and hospital categories in China. Economically developed southeastern coastal regions exhibit higher efficiency levels compared to central, western, and northeastern areas, where institutional and managerial bottlenecks hinder progress. Moreover, general hospitals often face structural inefficiencies due to misaligned resource allocation and functional positioning, while specialty hospitals demonstrate higher efficiency in resource utilization.

In Guangdong Province, one of China’s most economically dynamic and resource-rich regions, healthcare institutions face substantial challenges in achieving equitable and efficient service delivery. While some hospitals have demonstrated remarkable efficiency gains, others continue to struggle with resource redundancy and insufficient outputs. This raises critical questions: What are the underlying factors driving efficiency disparities among different hospital categories? How do these disparities evolve over time? And what measures can be implemented to optimize resource allocation and improve overall service efficiency?

In recent years, numerous studies have focused on the effects of these healthcare reform policies, demonstrating significant improvements in health service management and greater equity and balance in key areas such as chronic disease prevention. However, prominent disparities in service efficiency persist among regions and across different types of healthcare institutions, and practical challenges remain in implementing and continuously refining performance management (6, 7).

Service efficiency, as a key indicator for evaluating the performance of healthcare institutions, has become a focal point in the reform and management of health systems worldwide (8). In China, public hospitals serve as the core of the healthcare system, and their efficiency levels directly affect the accessibility, equity, and effective allocation of healthcare resources (9). Both domestic and international scholars have employed Data Envelopment Analysis (DEA), the Malmquist index, Analytic Hierarchy Process (AHP), and other methodologies to empirically measure the service efficiency of healthcare institutions at different levels and types. The results generally show that medical service efficiency in economically developed and southeastern coastal regions is significantly higher than that in central, western, and northeastern areas, indicating a highly uneven regional distribution. Some municipal and primary healthcare institutions experience redundant inputs and insufficient outputs. Based on large national samples and multidimensional data, research has also identified that the key variables influencing efficiency include not only resources such as manpower, equipment, and bed capacity, but also hospital management practices, performance assessment, policy incentives, patient structure, and the level of informatization (56). Notably, the national performance evaluation of tertiary public hospitals incorporates external quality assessment, patient safety, and process reengineering as core indicators, guiding healthcare institutions at all levels to continuously improve service efficiency and quality through PDCA cycles and information management tools (5). In addition, new models of digital healthcare, such as internet hospitals and intelligent triage systems, have also been shown to optimize medical processes, enhance patient experience, and improve operational efficiency (9).

In Guangdong Province, which stands out as China’s most economically dynamic, densely populated, and resource-rich region, improving the service efficiency of healthcare institutions is crucial for safeguarding public health and promoting the high-quality development of the health sector (6). In recent years, Guangdong has advanced the performance evaluation of public hospitals, deepened the implementation of medical alliances and hierarchical diagnosis and treatment systems, and increased investment in primary healthcare institutions, thus effectively enhancing the coordination and capacity of regional healthcare services. Empirical research has found that some areas and hospitals in Guangdong have achieved remarkable improvements in medical service efficiency, yet challenges such as regional development imbalances, sluggish efficiency gains in primary institutions, and fluctuations in resource utilization persist. Therefore, systematically reviewing and quantifying the dynamic evolution of service efficiency in healthcare institutions across Guangdong Province, and exploring its influencing factors, is of both theoretical and practical significance for optimizing regional resource allocation, improving overall service quality, and refining performance management systems. It also offers valuable insights and pathways for similar regions and policymakers nationwide.

## 2 Literature review

### 2.1 Research on methods for measuring healthcare service efficiency

Healthcare service efficiency serves as a critical metric for evaluating health system performance, with various measurement methodologies gaining widespread application in recent years. Data Envelopment Analysis (DEA) is particularly suitable for systems characterized by multiple inputs and outputs. It does not require a predefined production function and is well-adapted to nonparametric features, making it the most widely used approach for evaluating healthcare efficiency. DEA enables the assessment of technical efficiency, scale efficiency, and overall efficiency within a unified framework (10). As the application scenarios for DEA have expanded and model structures have improved, Bootstrap DEA has been increasingly used for robustness analysis, offering estimation advantages in situations with limited sample size or unknown data distributions (11).

In the two-stage DEA model, the first stage estimates efficiency values, while the second stage identifies the impact of environmental variables through Tobit regression or stochastic frontier analysis. Some studies have incorporated hospital size, regional characteristics, and workforce structure into the explanatory pathway, thereby enhancing the interpretability of efficiency outcomes (12, 13). Other studies have integrated principal component analysis and clustering techniques to aggregate input and output indicators, which improves the stability and clarity of the results (10). Furthermore, the network DEA model is suitable for scenarios involving complex structures and significant path dependence within organizational divisions. This approach extends the traditional “black box” evaluation toward a more traceable, stage-based evolution, where the input–output relationships among different operational nodes are explicitly modeled. Dynamic DEA constructs multi-period performance sequences, allowing for the assessment of evolutionary trajectories in time series and providing a technical pathway to observe the persistence and fluctuations of healthcare efficiency (14). In summary, the main efficiency evaluation methods commonly used in healthcare service research are summarized in Table 1.

**Table 1:** Overview of common efficiency evaluation methods for healthcare institutions.

Method type	Basic principle	Applicable scenario	Methodological features	Representative literature
Traditional DEA Model	Constructs a frontier via linear programming to compare relative technical efficiency	Evaluates the efficiency of multiple decision-making units (hospitals) at a single point in time	Does not require pre-specified functional forms; simple to implement but sensitive to outliers	(55)
Bootstrap DEA	Introduces bootstrapping on the DEA basis to estimate confidence intervals	Robustness testing for efficiency in small-sample scenarios	Improves robustness of estimation, enables statistical inference, computationally complex	(11)
Two-stage DEA + Tobit/SFA	First stage measures efficiency via DEA, second stage uses Tobit or SFA to explain differences	Analyzes the impact of environmental or organizational factors on efficiency	Helps identify drivers of efficiency, with enhanced explanatory power	(12, 13)
Network DEA (NDEA)	Constructs multi-node structures to reflect internal divisions of hospital processes	Suitable for complex-process healthcare institutions, such as multi-department hospitals	Captures inter-stage relationships and resource transformation; high modeling requirements	(12, 40)
Dynamic DEA	Introduces a temporal dimension to form a panel DEA model	Multi-period performance comparisons, long-term performance assessment	Evaluates efficiency changes over time; requires consistent time-series data	(14)
Quality-adjusted DEA	Incorporates service quality into output variables to build a quality-adjusted efficiency model	Focuses on both technical efficiency and healthcare quality performance	More comprehensively reflects real performance, aligns with policy evaluation needs	(13)
Stochastic Frontier Analysis (SFA)	Specifies a functional form to estimate technical efficiency and random error terms	Efficiency measurement scenarios sensitive to data noise	Distinguishes inefficiency from random error; suitable for statistical modeling	(10)
Multi-stage DEA	Divides the production process into multiple stages: input, intermediate output, and final output	Healthcare institutions with clearly defined processes or multiple subsystems	Better reflects medical logic, suitable for department-level evaluation	(12, 40)

International empirical evidence shows that multi-output efficiency assessments gain explanatory validity when clinical quality and access dimensions are explicitly integrated (15). Portuguese public hospital data indicate that operational efficiency can coexist with safety, appropriateness, timeliness and access, cautioning against purely cost–output evaluations and underscoring the value of quality-adjusted or multidimensional DEA specifications (15). This supports our inclusion of quality and workforce structure variables in the subsequent explanatory stage.

### 2.2 Regional disparities and hospital type heterogeneity in the efficiency of Chinese healthcare institutions

Regional disparities and hospital-type heterogeneity have consistently shaped the landscape of healthcare service efficiency in China. Efficiency levels across different regions and institutions are often constrained by technological, managerial, and institutional factors. Research indicates that healthcare service efficiency demonstrates a hierarchical geographic distribution, with the highest efficiency observed in Central China and the lowest in the Northeast; imbalances in regional technology are a principal source of efficiency divergence (16). Central and western regions are hampered by institutional and managerial bottlenecks, resulting in slow changes in total factor productivity and a widening efficiency gap between regions (17). Systematic differences are also evident among hospital types: rural traditional Chinese medicine hospitals are particularly affected by redundant resources and insufficient outputs, with sluggish improvements in service capacity (18). Similarly, substantial differences exist between general and specialty hospitals, with general public hospitals facing structural disadvantages; misalignments between resource allocation and functional positioning lead to their relatively low efficiency (19). Economically advanced regions, while improving their own service efficiency, also create positive spatial spillover effects for neighboring regions, thereby promoting collaborative improvements in regional healthcare efficiency (20). These efficiency differences are not the result of single-variable effects, but rather reflect the systemic interactions among regional economic foundations, hospital organizational structures, and levels of resource coordination.

### 2.3 Dynamic evolution of healthcare institution service efficiency

The dynamic evolution of healthcare institution service efficiency essentially reflects the combined effects of institutional adaptability, technological diffusion capacity, and governance synergy. The underlying mechanisms of efficiency changes are not only associated with the advantages and disadvantages of static resource allocation, but also reflect an institution’s capacity to adapt to external environments and update its internal management. With the advancement of performance assessment mechanisms, some regions and institutions develop path dependencies that facilitate efficiency improvements, while others are limited by institutional inertia and lags in technological diffusion, resulting in slow efficiency gains. Related studies indicate that healthcare service efficiency in China exhibits spatial agglomeration patterns, with “club convergence” occurring between high- and low-efficiency regions and limited cross-regional mobility (21). Urban and rural healthcare institutions follow divergent efficiency evolution paths: urban institutions maintain steady improvements through technological advancement, while rural institutions sustain technical efficiency but overall efficiency is constrained by resource bottlenecks (22). The internal positive spatial spillover effects within regions are increasingly pronounced, with efficiency convergence among geographically proximate healthcare institutions, reinforcing hierarchical regional structures (23). Reversals in technology, fiscal fluctuations, and managerial inefficiencies have also been confirmed as key contributors to efficiency declines (24). In the long run, the essence of efficiency evolution lies in organizational adaptation and institutional response; driven by both institutional governance and data analytics, dynamic efficiency analysis will become an essential tool for evaluating the effectiveness of healthcare reforms and conducting post-policy assessments.

Exogenous shock evidence reinforces the need for dynamic, distribution-sensitive efficiency tracking (25). Pre- versus intra-pandemic comparisons of Portuguese public hospitals show that systemic shocks can temporarily reconfigure efficiency trajectories while revealing underlying resilience differentials (25). This justifies our complementary use of Dagum inequality decomposition and kernel density methods to distinguish structural divergence from shock-induced perturbations when interpreting fluctuation, bimodality and partial recovery signals in Guangdong.

### 2.4 Empirical studies on the determinants of healthcare service efficiency

A solid economic foundation underpins improvements in efficiency; in the context of limited resource mobility, regional GDP and household income have a decisive impact on the service capacity of medical institutions (26). The structural scale of healthcare institutions and the configuration of professional staff affect both the intensity of service delivery and the rate of resource consumption. Hospitals at different levels exhibit significant differences in human capital alignment, technological coverage, and response speed (27). Improvements in healthcare efficiency cannot rely solely on increased material input; the design of internal incentive structures determines the marginal output from resource utilization. The long-term shaping of hospital operational objectives and service behaviors depends on the degree of coordination among performance evaluation methods, fiscal subsidy models, and management processes. Institutionalized subsidies, if not guided by results-oriented principles, often diminish the efficiency of resource allocation (28). Efficiency is not a one-dimensional optimization result but rather a trade-off between institutional and resource factors.

Hospital efficiency is closely related not only to workforce density but also to staff structure, degree of matching, and incentive mechanisms. The clarity of management philosophy and the ability to promote information flow and collaborative mechanisms across departments are key determinants of the smoothness of service processes. The mere establishment of healthcare information systems does not equate to the formation of true informatics capacity; whether such systems genuinely serve clinical processes or become an added burden is essential for evaluating their contribution to efficiency. If there is a lack of dynamic balance between exploration and exploitation behaviors within the organization, it often manifests as periodic fluctuations in efficiency metrics (29, 30). From an operational logic perspective, empirical research on healthcare efficiency is shifting from static indicator selection to the modeling of structural behavioral variables. The evaluation of managerial effectiveness, technological responsiveness, and institutional adaptability is becoming a key direction for future research.

### 2.5 Research on the performance appraisal system of Chinese public hospitals

The performance appraisal system for Chinese public hospitals is a national-level, systematic evaluation mechanism established to strengthen hospital governance, enhance operational efficiency, and improve service quality. Its core objective is to promote hospital management refinement, scientific decision-making, and institutionalization through assessment-driven improvement, supervision, and development. This system, spearheaded by the National Health Commission, has been fully implemented nationwide since 2019, and a relatively mature policy and implementation framework has been established.

In recent years, the performance appraisal system has played a crucial role in guiding hospitals toward behavioral optimization and improved resource allocation efficiency. With respect to indicator design, some studies emphasize the construction of multidimensional systems covering input, process, output, and outcome indicators, and use methods such as the analytic hierarchy process and entropy weighting to determine indicator weights, thereby enhancing objectivity and adaptability (31). Evaluation tools have gradually evolved from traditional scoring methods to intelligent models such as artificial neural networks, backpropagation algorithms, and DEA-Tobit combinations, which improve the ability to identify performance differences (19, 32). In terms of assessment logic, equal emphasis is placed on “process and outcome,” and the “structure-behavior-outcome” closed-loop framework is widely used, providing theoretical support for systemic reforms. Performance assessment now encompasses not only operational and financial efficiency but also increasingly integrates humanistic dimensions such as patient satisfaction, medical safety, and staff motivation, advancing toward a people-centered approach (33).

In evaluating the effects of reforms, existing literature notes that models such as the Sanming model in Fujian—which involve redesigning incentive structures, reconstructing governance frameworks, and linking payment methods—have significantly reduced medical costs and improved hospital operational efficiency, demonstrating the amplification effect of institutional reform and governance synergy on performance optimization (34). Regarding institutional improvement pathways, researchers generally believe that appraisal mechanisms should be adapted to local conditions and embedded within the internal incentive systems of hospitals, forming a closed loop of “assessment–feedback–incentive–behavioral adjustment” (35). Performance appraisal has become a key tool for the Chinese government in public hospital governance, and it continues to play a positive role in policy implementation and service capacity enhancement.

## 3 Materials and methods

### 3.1 Indicator selection and data sources

In constructing the evaluation indicator system, this study adhered to the principles of scientific rigor, systematic design, policy orientation, dynamism, and data accessibility. Based on the national monitoring indicator system outlined in the *Operational Manual for Performance Assessment of National Tertiary Public Hospitals (2023 Edition)*, relevant indicator data from the sampled hospitals for the years 2018–2022 were collected. An input–output indicator system was established, comprising 2 primary indicators, 5 secondary indicators, and 11 tertiary indicators, as detailed in Table 2.

**Table 2:** Construction of the evaluation indicator system for service efficiency of healthcare institutions in Guangdong Province.

Primary indicator	Secondary indicator	Tertiary indicator	Indicator description	Primary indicator
Medical Service Input	Human Resource Input	Number of Health Technicians (persons)	The sum of active health technicians (including physicians, pharmacists, nurses, technicians) in the assessment year	Positive
	Material Input	Number of Actual Open Beds	Actual number of open beds (units)	Positive
	Financial Input	Fiscal Appropriation Income (10,000 RMB)	Sum of general public budget appropriations, government fund appropriations, and state-owned capital operation budget appropriations	Positive
Medical Service Output	Revenue-Expenditure Structure	Proportion of Personnel Expenses (%)	Personnel expenses/medical activity expenses × 100%	Positive
		Asset-Liability Ratio (%)	Total liabilities/total assets × 100%	Negative
		Proportion of Medical Service Revenue (%)	Medical service revenue (excluding drug, consumables, examination, and test income) as a proportion of total medical revenue	Positive
		Energy Consumption per 10,000 RMB Income (tons of standard coal/10,000 RMB)	Total annual energy consumption/ total annual income × 10,000	Negative
	Cost Control	Outpatient Average Cost Increase (%)	(Current year average outpatient medical cost – previous year average outpatient medical cost)/previous year average outpatient medical cost × 100%	Negative
		Outpatient Average Drug Cost Increase (%)	(Current year average outpatient drug cost – previous year average outpatient drug cost)/previous year average outpatient drug cost × 100%	Negative
		Inpatient Average Cost Increase (%)	(Current year average inpatient medical cost – previous year average inpatient medical cost)/previous year average inpatient medical cost × 100%	Negative
		Inpatient Average Drug Cost Increase (%)	(Current year average inpatient drug cost – previous year average inpatient drug cost)/previous year average inpatient drug cost × 100%	Negative

The chosen time span of 2018–2022 is particularly relevant for capturing the dynamic changes in hospital service efficiency during a critical phase of healthcare reform in China. This period coincides with the full implementation of the national performance evaluation system for tertiary public hospitals, which began in 2019, providing a standardized framework for assessing hospital efficiency. Additionally, Guangdong Province experienced significant advancements in healthcare system modernization during this timeframe, including the expansion of medical alliances, the refinement of hierarchical diagnosis and treatment systems, and increased investments in primary healthcare institutions. The inclusion of data from 2018 allows for a baseline comparison prior to the widespread implementation of these reforms, while 2022 represents the most recent available data, enabling a comprehensive analysis of trends and impacts over 5 years. This selection ensures that the study captures both pre-reform conditions and post-reform outcomes, providing valuable insights into the evolution of service efficiency.

A stratified sampling approach was employed, adhering to principles of representativeness and generalizability, yielding a sample of 22 tertiary public hospitals in Guangdong Province. In terms of institutional composition, the sample included 10 general hospitals, 4 traditional Chinese medicine hospitals, 4 specialty hospitals, and 4 maternal and child health hospitals. Regarding administrative level, the sample comprised 10 provincial-level hospitals, 7 municipal-level hospitals, and 5 district/county-level hospitals. In terms of hospital classification, there were 20 Class A tertiary hospitals and 2 Class B tertiary hospitals. Therefore, the selected sample is considered to be representative of the tertiary public hospitals in Heilongjiang Province.

### 3.2 Research methods

#### 3.2.1 Data Envelopment Analysis (DEA)

Data Envelopment Analysis (DEA), a non-parametric evaluation technique rooted in linear programming, is utilized to assess the relative efficiency of Decision Making Units (DMUs) (36, 37). This approach was first proposed by Charnes, Cooper, and Rhodes in 1978, with its core idea being to construct an efficient frontier under conditions of multiple inputs and outputs to identify the efficient DMUs within the production possibility set (38). The efficient frontier represents the set of maximum possible outputs given certain inputs, or the minimum inputs required for a given set of outputs. DEA is widely used for evaluating production efficiency in fields such as healthcare, education, and finance (39–42), particularly in scenarios where there are multiple inputs and outputs and the specific form of the production function is difficult to determine. This method can reveal the efficiency levels of DMUs and distinguish the potential output levels under specific input conditions.

#### 3.2.2 Dagum Gini coefficient

The Dagum Gini coefficient, derived from the work of Camillo Dagum, is a significant extension and improvement of the traditional Gini coefficient. By employing a three-parameter model of probability distribution, this coefficient enables a more in-depth analysis and explanation of inequalities in income distribution (43). In the field of economics, the Dagum Gini coefficient effectively decomposes inequalities, revealing the distribution among different income groups and their contributions to overall inequality (44). Its application now extends far beyond economics, having been widely adopted in social sciences, public policy evaluation, and healthcare service research (45–47). For example, studies investigating the impact of globalization on income distribution in various countries have used the Dagum Gini coefficient to reveal both interregional and intraregional economic disparities and polarization. These studies demonstrate that the Dagum Gini coefficient is a powerful tool for providing in-depth inequality analysis, especially when evaluating policy effects.

#### 3.2.3 Kernel density estimation

Kernel density estimation is an important non-parametric statistical tool that estimates the probability density function of a variable through smoothing techniques, particularly when the underlying data distribution is unknown or complex. This method is widely applied across disciplines such as economics, ecology, and social sciences (48, 49). Specifically, in the field of healthcare services research, kernel density estimation is used to analyze the distributional characteristics and temporal changes of service quality. It can reveal regional disparities in service levels and identify dynamic trends, such as improvements or deteriorations in service quality. In China, with the deepening reform of the healthcare service system, kernel density estimation has become an essential tool for researchers analyzing regional disparities in healthcare services and assessing the equity of health resource allocation.

#### 3.2.4 Tobit regression model

The Tobit regression model, introduced by economist James Tobin, was developed to address censored data issues that standard regression models cannot effectively handle. In healthcare service research, censored or limited dependent variables are common, such as the prevalence of zero values representing non-utilization of certain services. The Tobit model is particularly suitable for such scenarios, as it estimates the effects for non-censored observations while also taking into account the information below the censoring threshold (50, 51). This model allows for effective estimation of economic behaviors when the dependent variable is limited, providing more precise analysis of influencing factors in the quality evaluation of healthcare services (52). The Tobit regression model continues to play a key role in various economic and social research fields. In health economics, it has been used to analyze the impact of health insurance on the utilization of medical services. In environmental economics, the model has been applied to evaluate the effects of environmental policies on firms’ compliance behaviors. These studies further validate the effectiveness of the Tobit model in handling censored or limited data, especially for policy impact assessment.

## 4 Empirical results and analysis

### 4.1 Efficiency levels and characteristics of medical institutions in Guangdong Province

Table 3 provides a comprehensive overview of the service efficiency levels and their dynamic changes among medical institutions in Guangdong Province from 2018 to 2022. A cross-sectional comparison reveals differences and trends in efficiency levels among various hospital types as well as individual hospitals. Over this five-year period, certain hospitals—such as Specialty Hospital 2 and Specialty Hospital 3—maintained the highest efficiency scores, with efficiency values of 1.000. In contrast, the efficiency level of General Hospital 1 declined sharply by 87.692%, ranking lowest among all hospitals.

**Table 3:** Service efficiency levels and rankings of medical institutions in Guangdong Province, 2018–2022.

Hospital category	Efficiency score					Mean	Mean rank	Growth (%)	Growth rank
	2018	2019	2020	2021	2022				
General Hospital 1	0.339	0.189	0.116	0.163	0.168	0.195	21	−87.692	22
General Hospital 2	0.443	0.230	0.272	0.194	0.472	0.322	17	9.006	9
General Hospital 3	0.223	0.207	0.276	0.153	0.394	0.251	19	68.127	1
General Hospital 4	1.000	0.650	0.518	0.385	0.906	0.692	13	−13.584	19
General Hospital 5	0.777	0.837	0.684	0.236	0.408	0.588	15	−62.755	21
General Hospital 6	0.628	1.000	1.000	1.000	1.000	0.926	6	40.173	3
General Hospital 7	0.155	0.210	0.181	0.147	0.206	0.180	22	28.333	5
General Hospital 8	0.234	0.437	0.291	0.308	0.239	0.302	18	1.656	10
General Hospital 9	0.356	0.806	0.372	0.412	0.584	0.506	16	45.059	2
General Hospital 10	0.936	0.782	0.728	0.603	0.759	0.762	12	−23.228	20
TCM Hospital 1	0.217	0.192	0.246	0.145	0.265	0.213	20	22.535	6
TCM Hospital 2	0.744	1.000	0.890	0.700	0.933	0.853	8	22.157	7
TCM Hospital 3	0.655	0.740	0.597	0.699	0.603	0.659	14	−7.891	16
TCM Hospital 4	1.000	1.000	1.000	0.495	0.916	0.882	7	−9.524	17
Specialty Hospital 1	1.000	1.000	0.934	0.994	0.891	0.964	4	−11.307	18
Specialty Hospital 2	1.000	1.000	1.000	1.000	1.000	1.000	1	0.000	11
Specialty Hospital 3	1.000	1.000	1.000	1.000	1.000	1.000	1	0.000	11
Specialty Hospital 4	0.848	1.000	0.534	0.694	1.000	0.815	9	18.650	8
Maternal & Child Hospital 1	0.448	1.000	0.994	0.677	0.732	0.770	11	36.883	4
Maternal & Child Hospital 2	1.000	1.000	0.984	0.742	1.000	0.945	5	0.000	11
Maternal & Child Hospital 3	1.000	0.723	0.687	0.626	1.000	0.807	10	0.000	11
Maternal & Child Hospital 4	1.000	1.000	1.000	1.000	1.000	1.000	1	0.000	11
Overall Mean	0.682	0.727	0.650	0.562	0.703	0.665	-	3.158	-
General Hospital Mean	0.509	0.535	0.444	0.360	0.514	0.472	4	1.059	4
TCM Hospital Mean	0.654	0.733	0.683	0.510	0.679	0.652	3	3.834	2
Specialty Hospital Mean	0.962	1.000	0.867	0.922	0.973	0.945	1	1.164	3
Maternal & Child Hospital Mean	0.862	0.931	0.916	0.761	0.933	0.881	2	8.059	1

From a longitudinal perspective, the overall mean increased slightly from 0.682 in 2018 to 0.703 in 2022, indicating a modest improvement in overall service efficiency. Further analysis of mean rankings and growth rankings in the table shows that the category of maternal and child health hospitals exhibited the most significant improvement in service efficiency, ranking first in average growth. By contrast, general hospitals showed relatively slow improvement, ranking fourth in average growth.

From the perspective of hospital categories, specialty hospitals had the highest average comprehensive efficiency among all types, with a mean value of 0.945. Their efficiency scores remained stable throughout the study period, with only a slight decrease observed in 2020. This indicates that specialty hospitals demonstrated high efficiency in resource utilization and service provision. Maternal and child health hospitals ranked second in average comprehensive efficiency (mean 0.881), and also showed the highest growth rate, reaching 8.059%, reflecting substantial progress in efficiency improvement. Traditional Chinese medicine (TCM) hospitals ranked third (mean 0.652), with the second-highest growth rate, indicating steady efficiency gains; although there was a decline in 2021, the overall trend suggests improvements in service efficiency for TCM hospitals. In contrast, general hospitals ranked lowest in both mean efficiency (0.472) and growth rate (1.059%), which may indicate room for improvement in terms of scale and technical efficiency, or the influence of other constraints.

**Figure 1:**
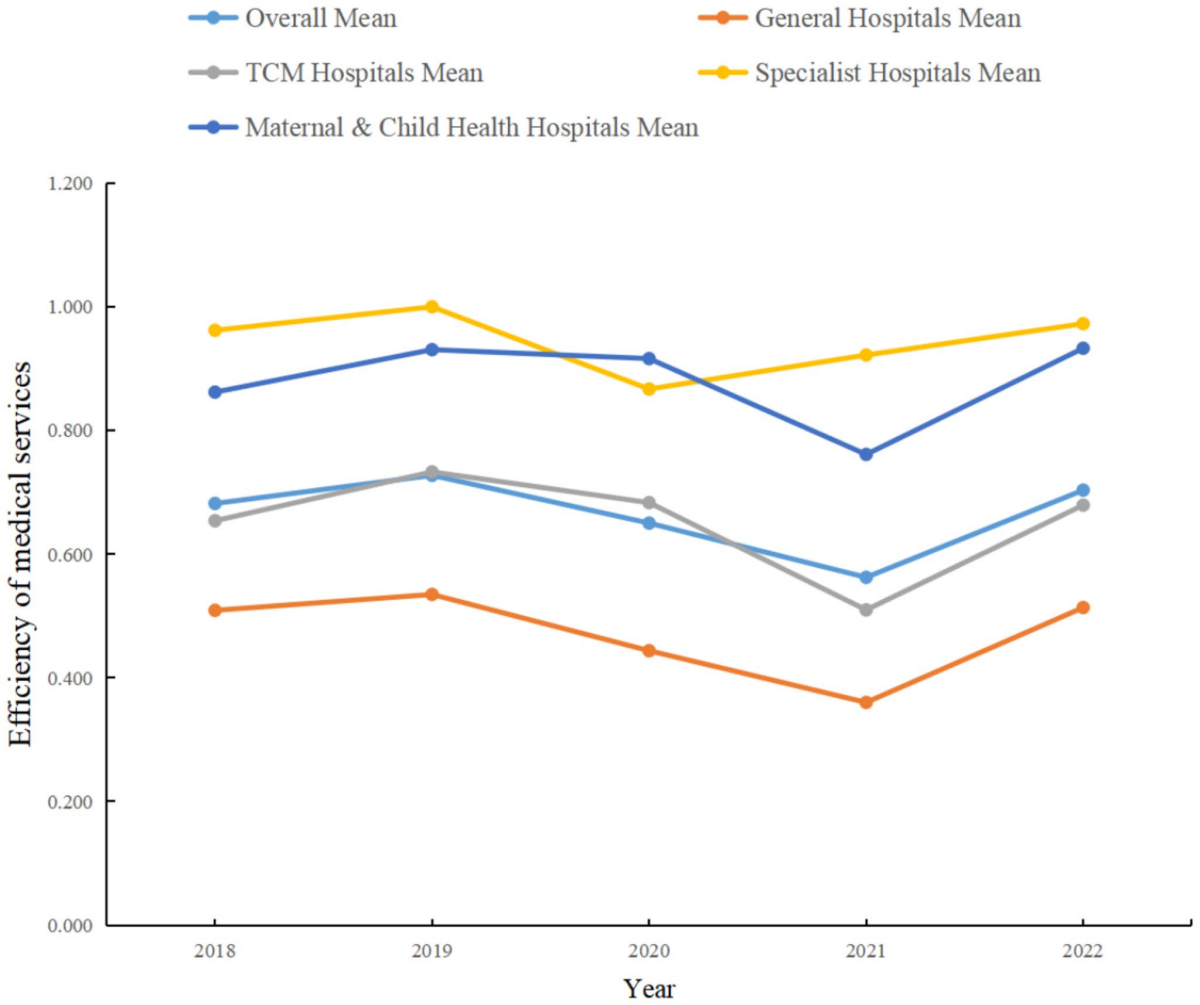
Mean comprehensive service efficiency by hospital category in Guangdong Province.

Overall, Figure 1 illustrates the evolution of service efficiency among different hospital categories, providing valuable evidence for hospital managers and policymakers to guide decision-making regarding improvement strategies and resource allocation. Understanding these trends can help inform future management practices, optimize service processes, and enhance the quality and efficiency of healthcare services.

**Figure 2:**
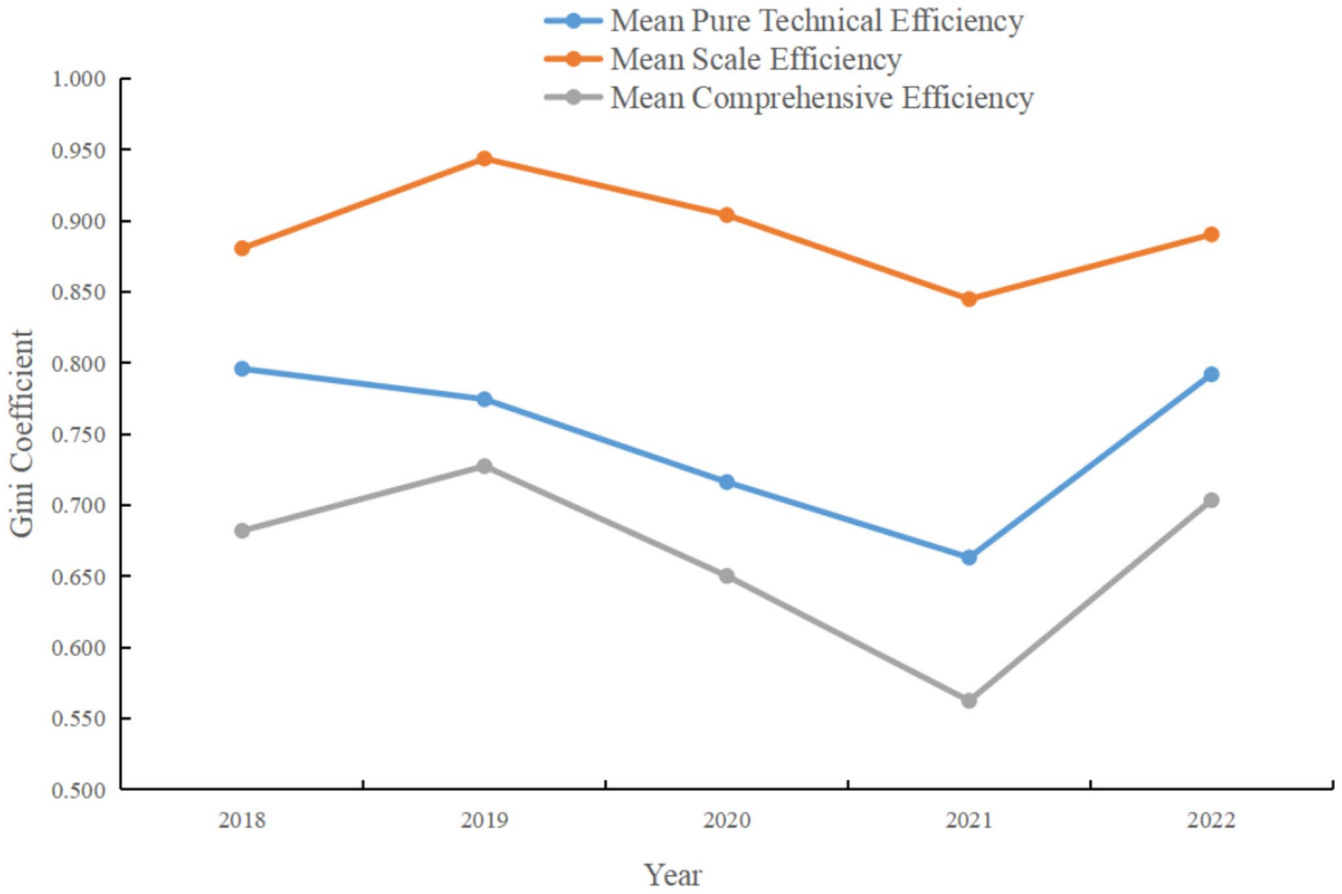
Mean pure technical efficiency, comprehensive efficiency, and scale efficiency of medical institutions in Guangdong Province.

As shown in Figure 2, the average value of pure technical efficiency was 0.796 in 2018, followed by declines in 2019 and 2020, dropping to 0.663 in 2021 before rebounding to 0.792 in 2022. This may reflect the volatility of technical practices and efficiency under changing external environments and internal management strategies. The average scale efficiency started at 0.881 in 2018, followed a relatively steady downward trend to 0.845 in 2021, then recovered to 0.890 in 2022. This suggests that scale adjustments among medical institutions during the study period may not have fully realized economies of scale. Comprehensive efficiency increased gradually from 0.682 in 2018 to 0.727 in 2019, but experienced notable declines in 2020 and 2021, ultimately rebounding to 0.703 in 2022. This trend suggests that while there was potential for efficiency gains, medical institutions may have faced adverse factors such as funding constraints, policy changes, or managerial challenges.

These findings indicate that the performance of medical institutions in Guangdong Province fluctuated in terms of pure technical efficiency, scale efficiency, and comprehensive efficiency, but overall showed signs of recovery. Researchers and policymakers should continue to monitor these trends and explore the underlying factors affecting efficiency changes, in order to develop targeted measures and promote sustained improvement. Additionally, since comprehensive efficiency is a function of both pure technical efficiency and scale efficiency, improvements in either will directly enhance overall efficiency. Therefore, medical institutions should focus not only on improving operational efficiency, but also consider appropriate expansion or contraction of scale to achieve economies of scale.

### 4.2 Analysis of efficiency differences among various types of tertiary public hospitals in Guangdong Province

To further elucidate the magnitude and sources of service efficiency differences among various types of medical institutions in Guangdong Province, this study employs the previously introduced Dagum Gini coefficient and subgroup decomposition method to measure efficiency disparities. The analysis is conducted by decomposing the four main hospital categories: general hospitals, traditional Chinese medicine (TCM) hospitals, specialty hospitals, and maternal and child health hospitals.

#### 4.2.1 Within-group differences among hospital categories

**Figure 3:**
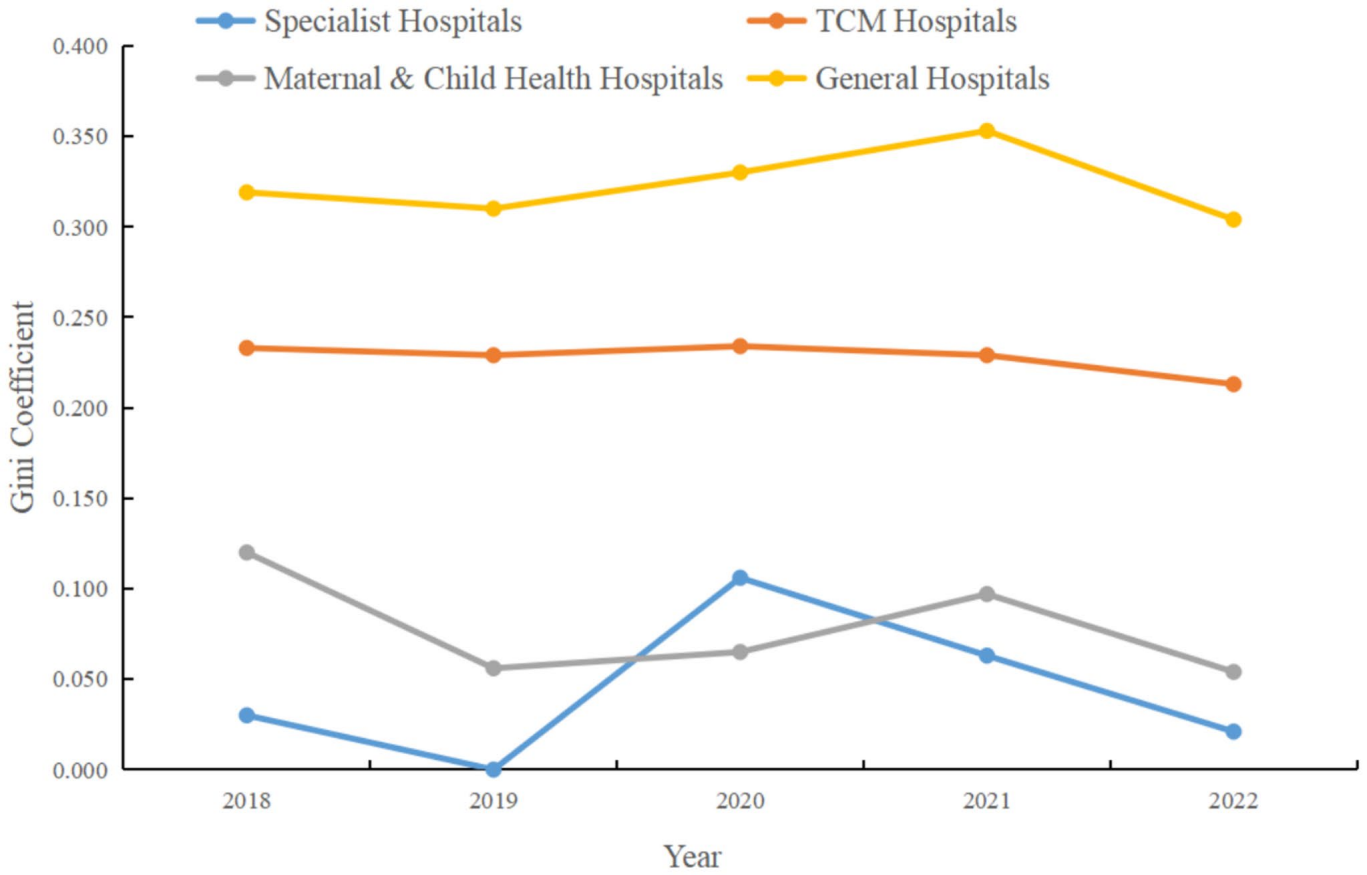
Within-group Gini coefficient for service efficiency by hospital category in Guangdong Province, 2018–2022.

Figure 3 presents the within-group Gini coefficients for each hospital category from 2018 to 2022. The Gini coefficient is a commonly used indicator for measuring inequality, with higher values indicating greater inequality. As shown in Figure 3, general hospitals exhibited relatively high Gini coefficients over the five-year period, peaking at 0.353 in 2021. This indicates the most pronounced service efficiency disparities within the general hospital category, potentially reflecting heterogeneity in scale, management, and resource allocation. In contrast, specialty hospitals had consistently low Gini coefficients, except for a modest rise to 0.106 in 2020, and even reached 0 in 2019, indicating a more uniform distribution of service efficiency within this category. The Gini coefficients for TCM hospitals and maternal and child health hospitals remained relatively stable throughout the period, with TCM hospitals ranging from 0.213 to 0.234, and maternal and child health hospitals between 0.054 and 0.120. This suggests smaller internal differences in service efficiency within these two categories, with TCM hospitals displaying slightly higher disparities than maternal and child health hospitals.

In summary, Figure 3 demonstrates the dynamic changes in intra-category service efficiency disparities among different hospital types. General hospitals show relatively large internal inefficiencies, specialty hospitals exhibit lower levels of inequality, while TCM hospitals and maternal and child health hospitals fall in between. These findings underscore the need for decision-makers and managers to pay attention to internal efficiency imbalances within hospitals, investigate their underlying causes, and implement targeted measures to reduce inequality and enhance overall service efficiency.

#### 4.2.2 Between-group differences among hospital categories

**Figure 4:**
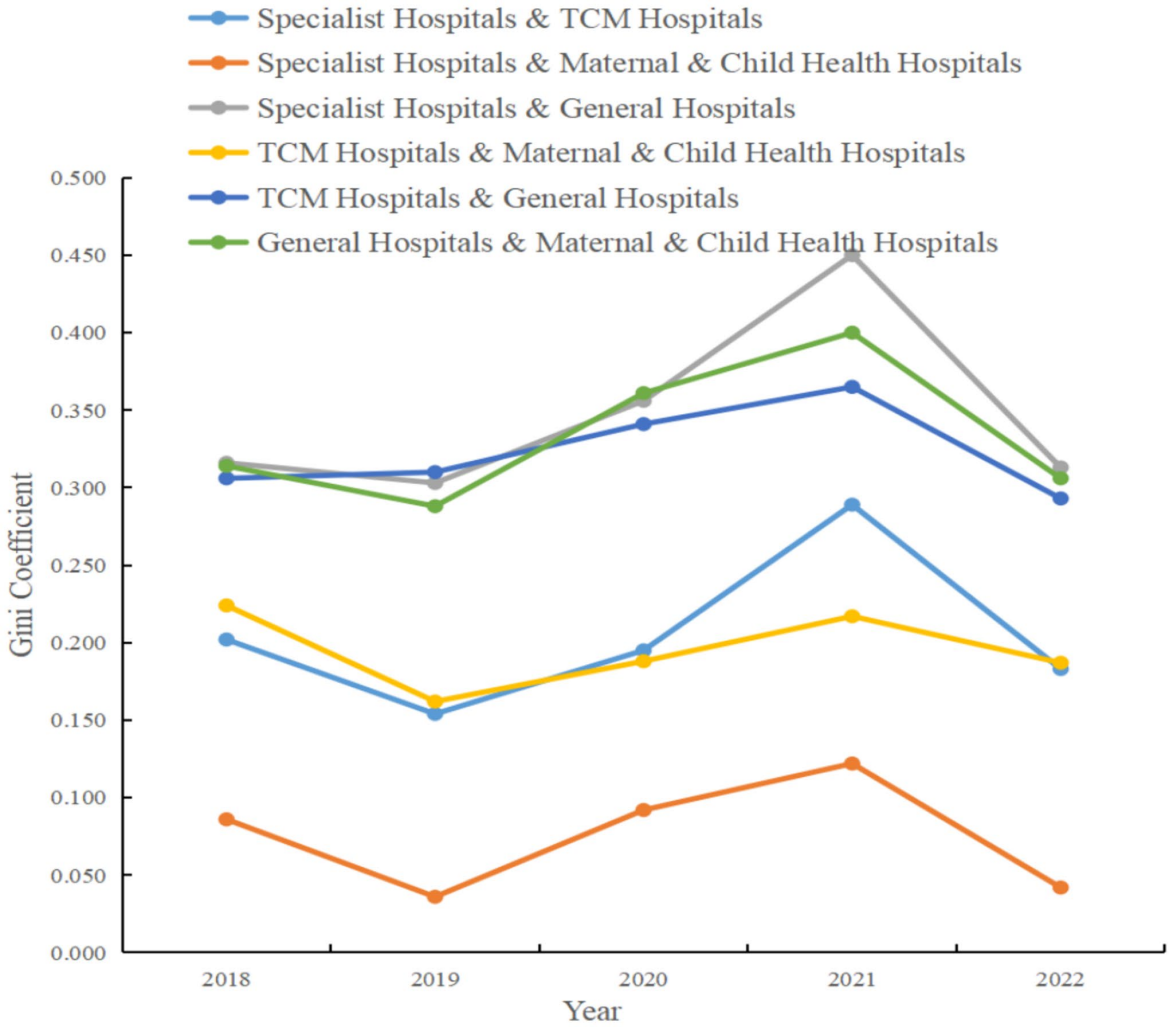
Between-group Gini coefficient for service efficiency by hospital category in Guangdong Province, 2018–2022.

The calculation of the Gini coefficient is based on the subgroup decomposition method, the results of which reflect the relative efficiency disparities between different hospital categories. Analysis of Figure 4 indicates that the Gini coefficients between specialty hospitals and general hospitals were relatively high throughout the time series, peaking at 0.450 in 2021, which signifies a significant increase in efficiency differences between these two categories. This may reflect increased specialization and efficiency in certain service areas among specialty hospitals, while general hospitals may face challenges related to the diversity of services and large-scale operations.

The between-group Gini coefficients for TCM hospitals and maternal and child health hospitals remained relatively stable during the study period, though they also increased to 0.217 in 2021, suggesting a widening disparity in efficiency distribution between these two categories for that year. Examining the coefficients between TCM hospitals and general hospitals, as well as between general hospitals and maternal and child health hospitals, reveals that both groups had relatively high efficiency disparities throughout the period, with peaks in 2020 and 2021. These patterns may be related to changes in management models, resource allocation, patient service scope, and levels of technical expertise across hospital types. Additionally, the Gini coefficient between specialty hospitals and TCM hospitals increased significantly in 2021 and decreased in 2022, which could be attributed to policy changes or market dynamics during those periods.

In summary, the analysis in Figure 4 highlights the substantial differences in service efficiency that exist among different hospital categories in Guangdong’s tertiary public hospitals. These disparities not only point to potential areas for optimization in resource allocation and management efficiency but also provide valuable data for future policy development aimed at improving the quality and efficiency of healthcare services.

#### 4.2.3 Sources and contributions of efficiency differences by hospital category

The decomposition of the Dagum Gini coefficient enables us to distinguish and quantify the sources of inequality, namely within-group differences (Gw), between-group differences (Gb), and transvariation density differences (Gt), as well as their respective contributions to the overall Gini coefficient. Analysis of the data in Table 3 reveals that the contribution rate of the between-group Gini coefficient (Gb) is the highest among the three sources in all years, reaching 68.053% in 2021. This underscores that differences in service efficiency between hospital categories are the primary source of overall inequality. Meanwhile, the contribution of the within-group Gini coefficient (Gw) is relatively low but remains stable over the time series, indicating that internal inefficiency accounts for a smaller share of overall inequality. The contribution of the transvariation density Gini coefficient (Gt) remains relatively constant throughout the period, possibly reflecting unevenness in efficiency distribution across different scales, with a comparatively minor impact on overall inequality.

In summary, the data in Table 4 reveal that overall inequality in service efficiency among tertiary public hospitals in Guangdong is mainly driven by differences between hospital categories. This finding is crucial for policymakers, as it suggests that efforts to improve healthcare service efficiency should focus particularly on reducing disparities between hospital types. Furthermore, these results provide a clear direction for future research—to further investigate the specific factors underlying inter-category efficiency differences and explore how effective management and resource allocation strategies can reduce such disparities.

**Table 4:** Sources and contributions of Dagum Gini coefficient decomposition for service efficiency differences.

Year	Gini coefficient				Contribution rate (%)		
	Overall Gini coefficient	Within-group Gini (Gw)	Between-group Gini (Gb)	Transvariation density Gini (Gt)	Contribution of Gw (%)	Contribution of Gb (%)	Contribution of Gt (%)
2018	0.255	0.063	0.145	0.046	24.772	56.986	18.242
2019	0.230	0.057	0.145	0.028	24.784	62.869	12.347
2020	0.272	0.062	0.168	0.042	22.919	61.749	15.331
2021	0.311	0.061	0.212	0.038	19.693	68.053	12.254
2022	0.236	0.056	0.150	0.030	23.741	63.692	12.567
Mean	0.2608	0.0598	0.164	0.0368	23.1818	62.6698	14.1482

### 4.3 Dynamic evolution of service efficiency levels in medical institutions in Guangdong Province

**Figure 5:**
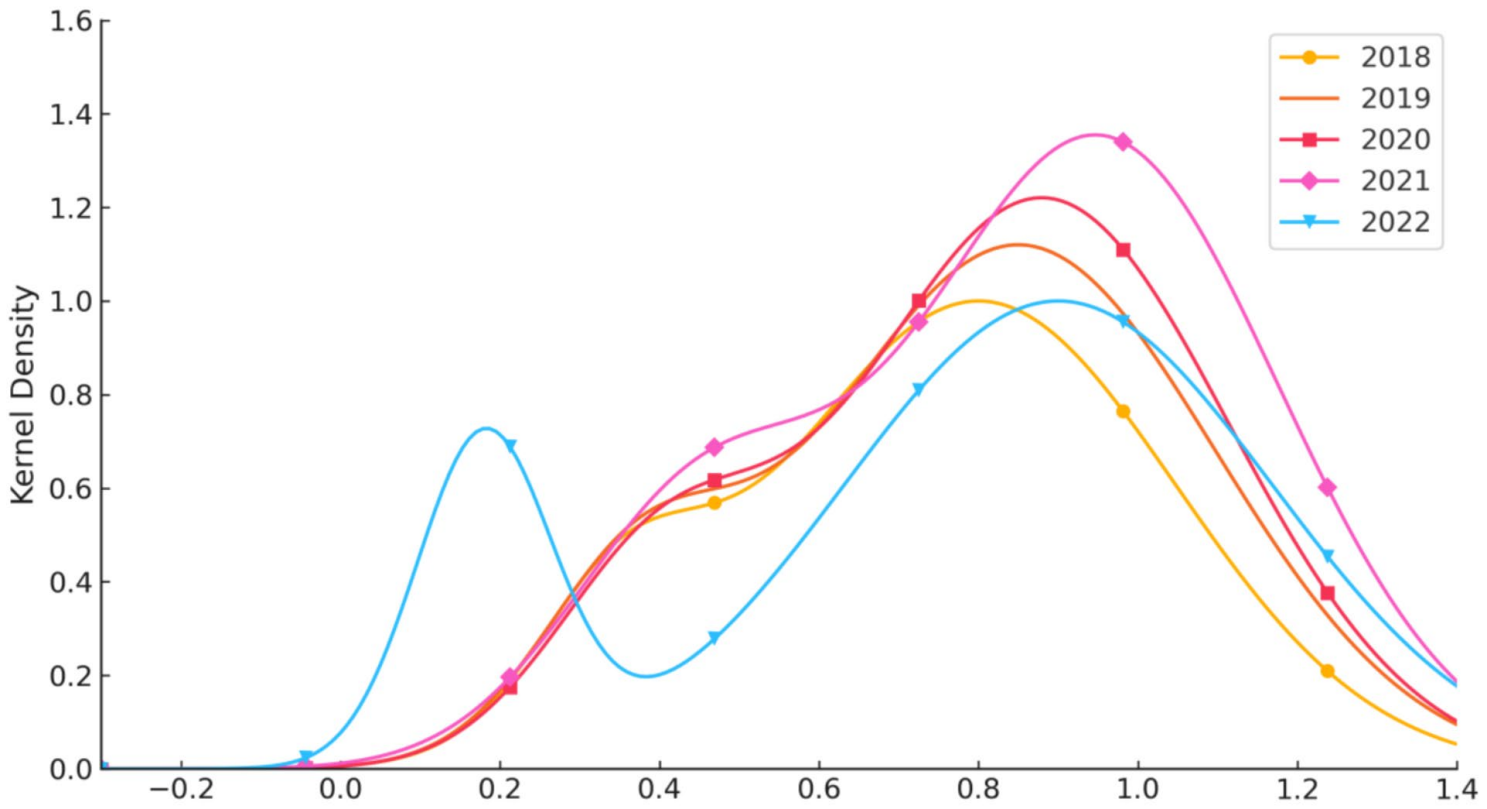
Two-dimensional kernel density estimation of the dynamic evolution of service efficiency levels in medical institutions in Guangdong Province, 2018–2022.

**Figure 6:**
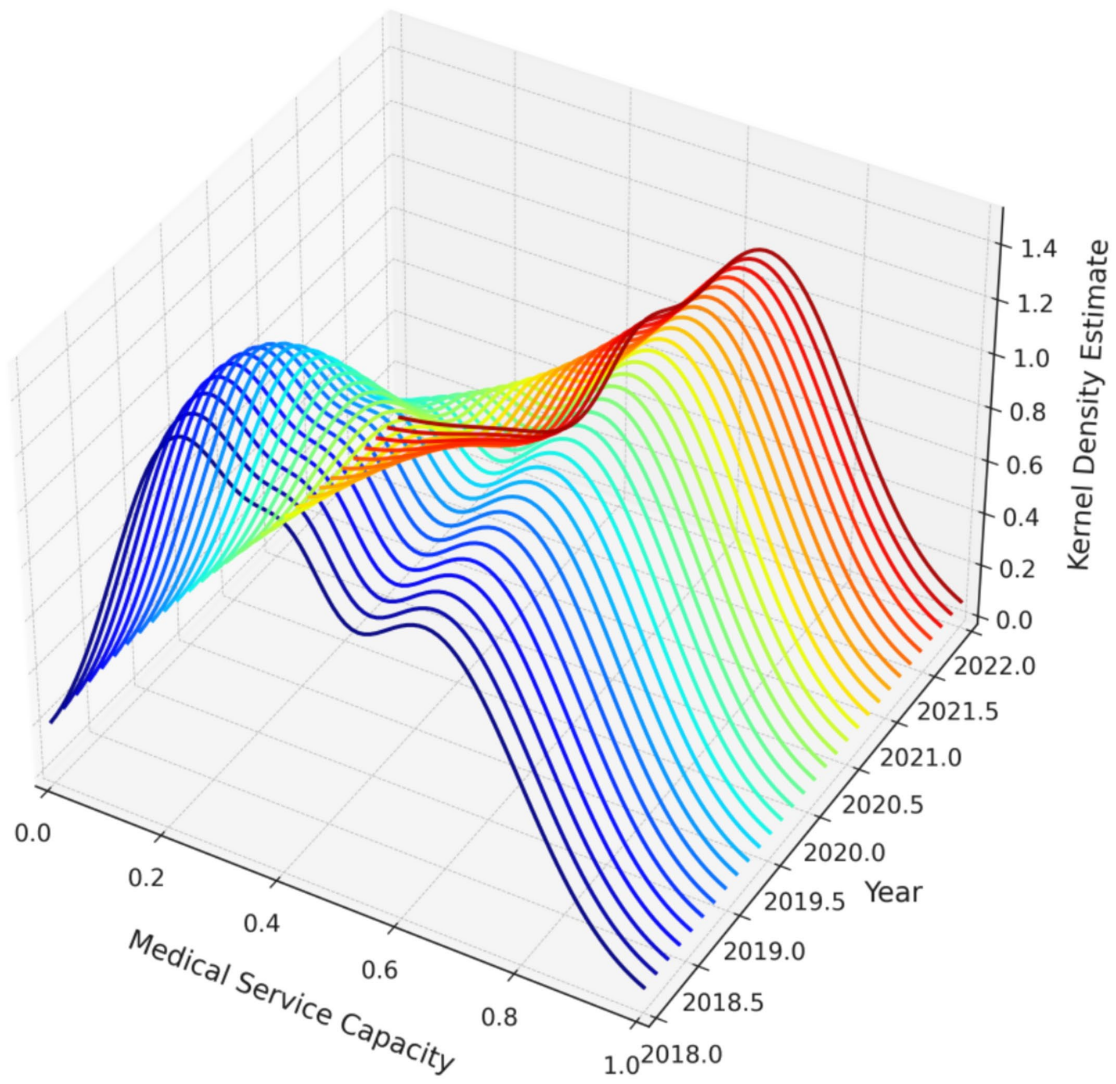
Three-dimensional kernel density estimation of the dynamic evolution of service efficiency levels in medical institutions in Guangdong Province, 2018–2022.

In this study, MATLAB R2021a software was used to perform Kernel density estimation of service efficiency levels among medical institutions in Guangdong Province from 2018 to 2022, employing the Gaussian kernel function. Both two-dimensional and three-dimensional Kernel density estimation plots are presented in Figures 5, 6, respectively. In terms of distribution, the efficiency levels exhibit both a main peak and a side peak in each year, indicating a bimodal distribution of service efficiency levels among medical institutions in Guangdong Province. The main peak reflects the efficiency levels of the majority of hospitals, while the side peak highlights the markedly different efficiency levels of certain hospital categories. This bimodal pattern indicates significant heterogeneity and an uneven distribution of service efficiency levels across hospital categories.

Furthermore, both the main and side peaks of the distribution show a leftward shift over the study period, suggesting an overall decline in service efficiency levels, or an increase in the number of hospital categories with lower efficiency. This leftward shift may be associated with factors such as uneven resource allocation, reduced accessibility of medical services, or changing patterns of healthcare demand. These findings provide important information for policymakers, indicating the need to focus on and improve those hospitals with persistently low service efficiency in order to enhance the overall efficiency and equity of medical services in Guangdong Province.

### 4.4 Determinants of service efficiency levels in medical institutions in Guangdong Province

#### 4.4.1 Variable definition and description

To further analyze the determinants of service efficiency among medical institutions in Guangdong Province, a Tobit regression model was employed, drawing on related research and official documents [(53, 54); Yang et al., 2022]. The comprehensive efficiency level of medical institutions in Guangdong Province from 2018 to 2022 was taken as the dependent variable. Twelve explanatory variables were selected to examine their impact on service efficiency: the proportion of special medical services, average appointment rate, mortality rate of low-risk patients, proportion of day surgeries among elective surgeries, and others. Detailed definitions of each variable are provided in Table 5.

**Table 5:** Variable definitions for determinants of service efficiency levels in medical institutions in Guangdong Province.

Variable type	Variable name	Variable description
Dependent	Comprehensive Efficiency	The average comprehensive service efficiency level of medical institutions in Guangdong Province, 2018–2022 (as derived above)
Independent	Proportion of Special Medical Services (%)	The percentage of revenue from special demand medical services (such as high-end or personalized care) in total medical revenue
	Average Appointment Rate (%)	The percentage of patients treated via appointment scheduling in the total daily patient population
	Mortality Rate of Low-Risk Group (‰)	The mortality rate (per thousand) among patients classified as low-risk within the hospital
	Proportion of Day Surgeries among Elective Surgeries (%)	The percentage of day surgeries (patients discharged on the same day of surgery) in all scheduled elective surgeries
	Average Daily Inpatient Workload per Physician (%)	The average number of inpatients managed daily per physician as a percentage of their total capacity
	Proportion of Surgical Patients among Discharges (%)	The percentage of discharged patients who underwent surgical procedures
	Proportion of Grade IV Surgeries among Discharges (%)	The percentage of discharged patients who underwent Grade IV surgeries
	Number of Pharmacists per 100 Beds	The number of pharmacists per 100 hospital beds
	Proportion of Health Technicians (%)	The percentage of health technicians (including doctors, nurses, and technicians) among total staff
	Proportion of Health Technicians with Intermediate or Higher Titles (%)	The percentage of health technicians with intermediate or higher professional titles among all health technicians
	Proportion of Anesthesiologists, Pediatricians, Intensivists, Pathologists, and TCM Physicians (%)	The percentage of these specialized physicians among all doctors
	Doctor-Nurse Ratio (%)	The ratio of physicians to nurses in the hospital

#### 4.4.2 Analysis of influencing factors

The Tobit regression results presented in Table 6 indicate that the intercept term is significantly positive (2.825, *p* < 0.01), which may reflect an overall positive baseline level of service efficiency among medical institutions in Guangdong Province. The mortality rate of low-risk patient groups (‰) has a significant positive effect on service efficiency (0.745, *p* < 0.01), suggesting that institutions with lower mortality rates in low-risk cases tend to perform better in terms of efficiency. This may be because effective management and treatment of low-risk cases are closely related to efficient service operations.

**Table 6:** Tobit regression coefficients for determinants of service efficiency.

Item	Coefficient
Intercept	2.825** (2.854)
Proportion of Special Medical Services (%)	−0.014 (−0.326)
Average Appointment Rate (%)	−0.005* (−2.214)
Mortality Rate of Low-Risk Patient Group (‰)	0.745** (3.735)
Proportion of Day Surgeries among Elective Surgeries	0.239 (0.958)
Average Daily Inpatient Workload per Physician	1.243 (1.541)
Proportion of Surgical Patients among Discharges (%)	−0.014 (−1.852)
Proportion of Grade IV Surgeries among Discharges (%)	−0.000 (−0.023)
Number of Pharmacists per 100 Beds	−0.013 (−0.938)
Proportion of Health Technicians (%)	−0.023* (−2.184)
Proportion of Health Technicians with Intermediate or Higher Titles (%)	−0.014* (−2.225)
Proportion of Anesthesiologists, Pediatricians, Intensivists, Pathologists, and TCM Physicians (%)	−0.001 (−0.757)
Doctor-Nurse Ratio (%)	−0.005 (−0.477)
log(Sigma)	−1.826** (−12.110)
Sample Size	22
Likelihood Ratio Test	*χ*2 (12) = 29.254, *p* = 0.004
McFadden R^2^	2.575

Dependent variable is comprehensive efficiency (OE); *p < 0.05, **p < 0.01; values in parentheses are *z*-values.

In addition, the average appointment rate (%), the proportion of health technicians (%), and the proportion of health technicians with intermediate or higher professional titles (%) all have significant negative effects on service efficiency at the 5% significance level (coefficients of −0.005, −0.023, and −0.014, respectively). This may indicate that these factors pose certain obstacles to improving service efficiency. The negative coefficients may reflect that a higher proportion of special medical services, a higher appointment rate, and a higher proportion of highly qualified health technicians do not always translate into greater efficiency. This could be related to improper resource allocation or suboptimal management.

Meanwhile, other explanatory variables, such as the proportion of special medical services, proportion of day surgeries among elective surgeries, average daily inpatient workload per practicing physician, proportion of surgical patients among discharges, proportion of Grade IV surgeries among discharges, number of pharmacists per 100 beds, and the proportion of anesthesiologists, pediatricians, intensivists, pathologists, and TCM physicians, were not significant in the model. This does not mean these variables are unimportant, but rather that, after controlling for other factors, their impact on service efficiency was not statistically significant.

The log (Sigma) term in the model is significantly negative (−1.826, *p* < 0.01), indicating relatively low variability in the model residuals, which enhances the credibility of the estimation results. The likelihood ratio test shows that the overall model is statistically significant (*χ*^2^ (12) = 29.254, *p* = 0.004), and the McFadden R^2^ is 2.575, indicating that the model has a certain degree of explanatory power.

## 5 Discussion and recommendations

### 5.1 Discussion

First, regarding the overall performance and trends of service efficiency among healthcare institutions in Guangdong Province, data analysis reveals that from 2018 to 2022, although there was a slight upward trend in overall service efficiency, substantial differences persisted between different hospital categories. Service efficiency improvements in general hospitals were relatively constrained, potentially attributable to the combined pressures of scale expansion and technological progression. In contrast, maternal and child health hospitals exhibited the most significant gains in efficiency, which may be attributable to their focused service scope, flexible management models, and strong policy support.

Second, the analysis of intra-hospital efficiency differences revealed considerable inequality within general hospitals, reflecting disparities in resource allocation, management capacity, and service processes among individual hospitals. The internal efficiency distribution of specialty hospitals was relatively uniform, likely due to their more specialized service fields and standardized processes. Furthermore, Gini coefficient analysis indicated that efficiency differences among traditional Chinese medicine hospitals and maternal and child health hospitals were relatively small, possibly because of their balanced service structures and management strategies.

Finally, in terms of the dynamic evolution of service efficiency, the bimodal distribution observed in kernel density estimation may indicate a clear divide between groups of high-efficiency and low-efficiency hospitals. The Tobit regression analysis showed that lower mortality rates among low-risk patient groups had a positive impact on service efficiency, whereas other factors such as average appointment rate exerted negative effects. This suggests that, while striving to improve service efficiency, it is also essential to balance the optimal allocation of healthcare resources and the rational design of service processes.

### 5.2 Recommendations

#### 5.2.1 Focus on structural efficiency shortcomings in general hospitals and promote refined stratified reform

Among all hospital categories, general hospitals consistently ranked lowest in both average efficiency and growth rates. The Dagum Gini coefficient indicated significantly greater intra-group efficiency disparities in general hospitals than in other categories, with a Gini coefficient reaching 0.353 in 2021. Technical efficiency and scale efficiency showed pronounced fluctuations, and some hospitals recorded efficiency levels consistently below the industry average for several consecutive years. The kernel density estimation identified a low-efficiency group mainly composed of general hospitals, highlighting systemic structural obstacles in resource allocation, management mechanisms, and technology diffusion. Some hospitals failed to effectively transform inputs into outputs, leading to both resource redundancy and insufficient service provision.

It is therefore crucial to promote the transformation of general hospitals toward refined and stratified governance. A stratified assessment mechanism based on functional positioning should be established, categorizing regional general hospitals into basic, extended, and referral types, with corresponding resource structures, evaluation indicators, and management authorities. Performance management should shift from uniform standards to differentiated guidance, emphasizing diagnostic efficiency and per capita output, with enhanced operational tracking supported by information technology. For hospitals with persistently low efficiency, a governance mechanism linking external oversight with internal management should be implemented, clearly defining efficiency accountability at the department and middle-management levels. Structural restructuring should break the inertia of “administrative-driven scale expansion,” and stimulate the hospital’s internal impetus for efficiency improvement.

#### 5.2.2 Optimize the horizontal and vertical linkage of performance assessment, and reconstruct the cross-category collaborative evaluation system

Results from the Dagum Gini decomposition indicate that inter-group differences were the dominant source of inequality in healthcare service efficiency in Guangdong Province between 2018 and 2022, with an average contribution rate as high as 62.67%. The efficiency gap between specialty hospitals and general hospitals was the most pronounced among all category pairs, with a Gini coefficient of 0.450 in 2021. Currently, performance assessments are implemented in a stratified manner at the hospital level, overlooking the combination of horizontal interactions across categories and longitudinal trends over time, which hampers the timely detection and feedback of cross-category efficiency changes.

There is a need to reconstruct the linkage dimensions of the assessment system, establishing a collaborative evaluation framework based on the triad of “category–region–time.” Core evaluation indicators should be mapped across different hospital categories to enhance both coordination and comparability, and to accommodate the dynamic flow of healthcare resources between institutions. Performance feedback mechanisms should incorporate modules for early warning of efficiency anomalies and trend deviation diagnostics, identifying intervention points for hospitals with sustained low efficiency, and employing reward–penalty linkages to guide the adjustment of service strategies. At the same time, data platform integration should be advanced to achieve comprehensive connectivity of performance information, from individual points to system-wide chains, thus enhancing institutional responsiveness and regulatory effectiveness, and providing policymakers with a basis for dynamic governance.

#### 5.2.3 Develop a differentiated resource allocation mechanism based on efficiency performance to guide resources toward high-performing units

The Tobit regression results show that overall hospital efficiency is closely related to human resource structure and service models, but resource input is not always proportional to efficiency output. Some high-investment hospitals exhibited relatively low efficiency, reflecting inadequacies in the current logic of fiscal and resource allocation to effectively identify performance differences. Existing input mechanisms are often based on institutional type and grade to set baseline allocations, without fully leveraging efficiency performance as a positive guide for resource acquisition.

The resource allocation mechanism should be restructured to use performance outcomes as a key basis for dynamic adjustment. A differentiated allocation formula for fiscal subsidies, staffing quotas, and special funds should be established, rationally incorporating efficiency indicators and performance rankings so that high-efficiency hospitals receive stronger resource support and development prospects. A positive feedback support system should be constructed to reinforce the linkage between efficiency and resources, enhancing the incentive sensitivity of efficiency-oriented management. Hospitals should be encouraged to concentrate resources on core output processes, and avoid administratively driven pursuit of indicators and non-productive expansion.

#### 5.2.4 Promote synergistic improvement of technical and scale efficiency

Technical efficiency and scale efficiency among healthcare institutions in Guangdong Province fluctuated asynchronously during the study period, indicating that some hospitals did not achieve simultaneous improvements in technical capabilities as resources expanded. If scale expansion is not accompanied by process optimization and technological integration, it is likely to lead to rising management costs and diminishing marginal returns. It is necessary to strengthen the pre-evaluation of efficiency in hospital expansion strategies and clarify the functional relationship between scale boundaries and resource returns. While improving scale efficiency, pathways for enhancing technical efficiency should be developed in parallel. The application of information systems should be deepened and data-driven operational decision-making mechanisms promoted to improve organizational capacity for managing complex resource portfolios. The introduction of intelligent technologies in non-clinical management, logistics coordination, and financial control can further enhance the human and financial efficiency of unit outputs. Through systematic collaborative improvement, the goal is to shift from partial optimization to systemic effectiveness.

## 6 Conclusion

Using DEA, Dagum Gini decomposition, kernel density analysis and Tobit regression for 22 tertiary public hospitals in Guangdong (2018–2022), we found: (1) Persistent inter-category heterogeneity—specialty hospitals were consistently most efficient, maternal & child hospitals improved fastest, TCM hospitals rebounded after a dip, and general hospitals stayed lowest in level and growth; (2) Overall inequality was structurally driven by between-group gaps (≈62.67% contribution on average) with pronounced within-group dispersion concentrated in general hospitals (peak Gini 0.353); (3) A stable bimodal distribution with leftward shifts in some years revealed a persistent low-efficiency cluster dominated by general hospitals; (4) Lower low-risk mortality was positively associated with efficiency, while higher average appointment rate, higher share of health technicians, and higher share with senior titles were negatively associated, indicating possible human resource and process misalignment. Prioritize refined, stratified governance of general hospitals; rebuild cross-category and temporal performance linkages to narrow dominant between-group gaps; embed efficiency-based differentiated resource allocation; and pursue synchronized improvement of technical and scale efficiency through data-driven process optimization and prudent capacity adjustments.

Future research should extend multi-level, multi-province samples; apply dynamic/network productivity metrics; integrate richer quality and patient-centered outcomes; incorporate spatial/convergence models for spillover assessment; and unpack micro-mechanisms of workforce structure, digital process integration, and incentive alignment.

## Data Availability

The original contributions presented in the study are included in the article/supplementary material, further inquiries can be directed to the corresponding authors.
